# Association between smoking status and handgrip strength in Korean male adults: based on Korea National Health and Nutrition Examination Survey 2016–2019

**DOI:** 10.3389/fmed.2023.1212946

**Published:** 2023-11-27

**Authors:** Eunbyul Cho, Hi Sun Soh, Jae-Ryun Lee, Jieun Yun, Woo Kyung Bae, Hyejin Lee

**Affiliations:** ^1^Department of Family Medicine, Seoul National University Bundang Hospital, Seongnam-si, Republic of Korea; ^2^Department of Family Medicine, Seoul National University Hospital, Seoul, Republic of Korea; ^3^Department of Pharmaceutical Engineering, Cheongju University, Cheongju-si, Republic of Korea; ^4^Health Promotion Center, Seoul National University Bundang Hospital, Seongnam-si, Republic of Korea; ^5^Department of Family Medicine, School of Medicine, Seoul National University, Seoul, Republic of Korea

**Keywords:** handgrip strength, smoking, non-smokers, ex-smokers, smokers

## Abstract

**Background:**

Smoking is a well-known risk factor of frailty. Handgrip strength (HGS) is highly representative of muscular strength and is used in the diagnosis of frailty; however, the relationship between smoking and HGS is not clear. We evaluated the relationship between smoking status and HGS.

**Objectives:**

This study aimed to evaluate the association between HGS and smoking status.

**Methods:**

We enrolled adult males between the ages of 19 and 80 years who participated in the Korea National Health and Nutrition Examination Survey VII–VIII. A chi-square test and ANOVA were performed to compare the mean handgrip strength (mean HGS) between non-smokers, ex-smokers, and current smokers. Logistic regression analysis was performed to determine the association between the smoking status and mean HGS, and additional analyses were performed by dividing subgroups by age.

**Results:**

A total of 7,649 participants were analyzed. When the mean HGS and mean dominant HGS were compared according to smoking status, HGS was higher in the right hand (value of *p* = 0.03) and left hand (value of *p* < 0.001) in the order of current smokers, ex-smokers, and non-smokers. Comparing HGS of stronger hands, the mean HGS ex-smokers [aOR, (95% confidence interval): 0.61 (0.46–0.82)] and current smokers: 0.55 (0.38–0.78) was higher than that of non-smokers. When subgroup analysis was performed according to age, current smokers aged >60 years had a higher grip strength than non-smokers.

**Conclusion:**

Current smokers had a stronger mean HGS than that of ex-smokers and non-smokers. Current smokers older than 60 years appeared to have a stronger mean HGS than ex-smokers and non-smokers of the same age group.

## 1 Introduction

Smoking poses a significant risk to global health, resulting in the premature deaths of six million individuals worldwide each year (1). It is a known contributor to cardiovascular diseases, including atherosclerosis, stroke, and ischemic heart disease (2). Furthermore, smoking increases the risk of cancer, including lung, gastric, liver, bladder, and cervical cancers (3). Smoking is also associated with mental health conditions such as schizophrenia, bipolar disorder, anxiety disorder, and depression (4, 5). In 2017, 38.1% of adult men in Korea and 6.0% of women were smokers (6).

Smoking is also a risk factor for frailty and sarcopenia (7). Frailty is a state of increased vulnerability to poor resolution of homeostasis following a stress (8, 9). Smoking can cause frailty by increasing the risk of physical problems and disabilities (10, 11). In a meta-analysis of the association between smoking and frailty, smoking might play a role in the pathogenesis of frailty and the association may be explained by multifactorial effect of wide range of organs and tissues, as well as chronic inflammation causing muscle wasting, weight loss, exhaustion, weakness and slow gait speed (12). Sarcopenia, a systemic skeletal muscle disorder characterized by loss of muscle mass and function, is a major component of frailty (13, 14). Diminished muscle strength is associated with decreased function (8, 15), increased all-cause mortality (16, 17) and serves as a risk factor for incident cardiovascular disease (17). Therefore, preventing the loss of muscle mass due to biopsychosocial factors or behaviors is of utmost importance, with smoking being one potential factor that could influence muscle strength (18). Nevertheless, studies examining the association between sarcopenia and cigarettes smoking remain controversial (19, 20).

Sarcopenia is considered an indicator of the development of frailty (21). Handgrip strength (HGS) is highly correlated with the overall muscle strength of the body (22). Measuring HGS is one of the easiest methods for measuring sarcopenia (23), and has been used as important index for diagnosing sarcopenia recently. This is due to the fact that low HGS is a clinical marker of poor mobility and proves to be a superior predictor of clinical outcome of low muscle mass (24–26). Moreover, HGS is a useful indicator for general health status, early all-cause mortality, cardiovascular mortality, disability, neural morbidities, functional declines, and mobility limitations (27, 28).

To the best of our knowledge, the relationship between smoking and HGS is unclear. Only a few studies have examined the relationship between smoking and grip strength. A previous study reported that generalization was difficult because of the small number of participants (29) and generation (18). Furthermore, One out of every three Korean males is a smoker, and among them, there are approximately 17% elderly smokers aged 65 and above. It is necessary to investigate the association between smoking and grip strength in order to develop intervention strategies for elderly smokers. Therefore, in this study, we aim to investigate the relationship between smoking and HGS in adult males using data from the 2016–2019 Korea National Health and Nutrition Examination Survey (KNHANES), a nationwide survey.

## 2 Materials and methods

### 2.1 Participants

**Figure 1:**
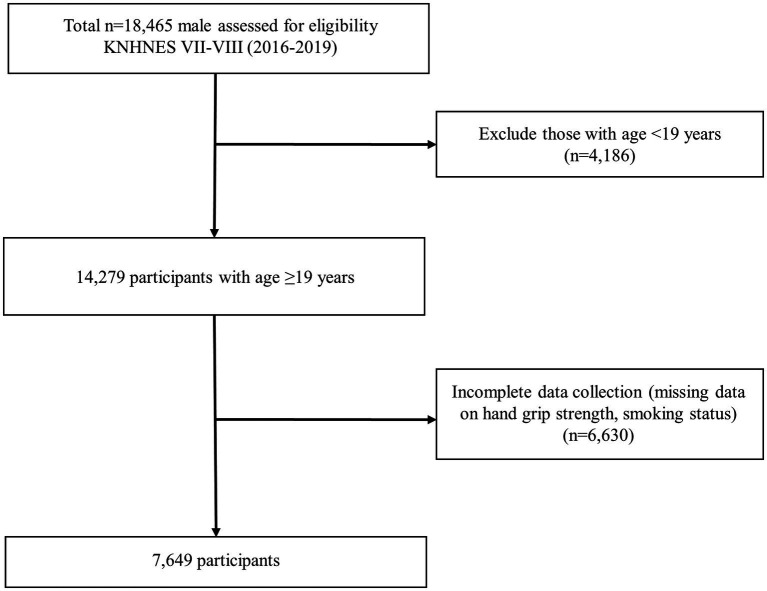
Overview of participants.

This study was conducted with adult males aged 19 years or older who participated in the 7th and 8th surveys of KNHANES and measured grip strength. KNHANES is a cross-sectional, nationally representative survey conducted by the Korea Centers for Disease Control and Prevention (KCDC). The survey collected demographic, socioeconomic, medical, and dietary information. KNHANES participants were selected using multistage stratified cluster sampling based on sex, age, and geographic area. In Korea, there is a tendency for women to conceal their smoking habits. Women are six times more likely to hide their smoking compared to men, and they also tend to underreport their smoking (30). Due to these factors, the data on female smoking is considered less reliable. Additionally, a distinct metabolism in genders differently affects sarcopenia development since men and women have different body compositions after puberty and sex hormones play a crucial role in this process (31). Since the female estimation showed moderate heterogeneity in meta-analysis of smoking status and sarcopenia (18), we included only men. Of the initial 18,465 subjects, 14,279 were aged 19 or older. Among them, 7,800 people were included, excluding those who did not respond to the survey on smoking. The grip strength test was conducted in three rounds, and 7,649 of the 7,800 people who performed all three grip strength test were selected as the final study participants (Figure 1).

### 2.2 Main variables

**Figure 2:**
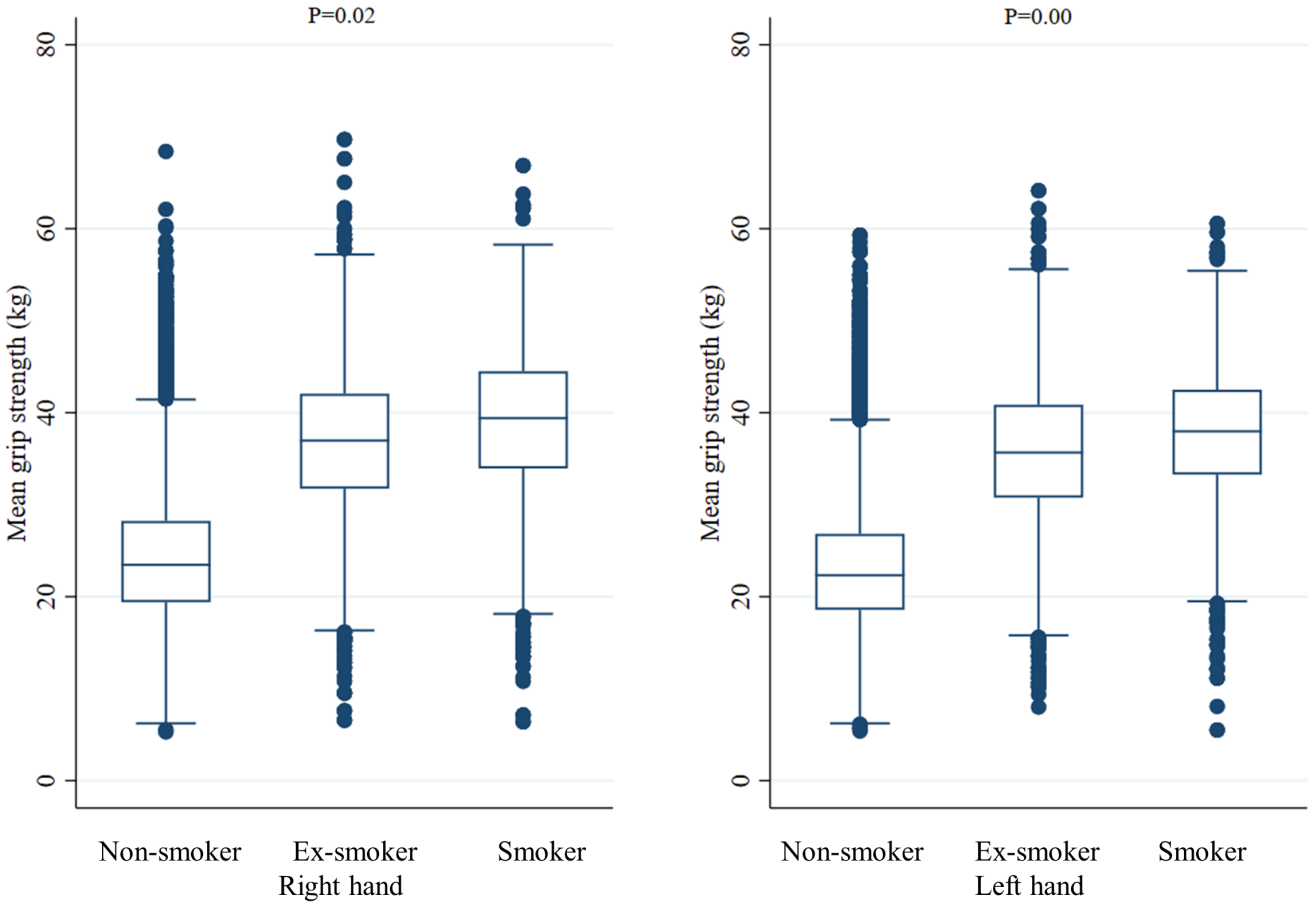
Mean handgrip strength according to smoking status. The middle horizontal line represents the median level. Whiskers go from minimum to maximum values with 1st and 3rd quartile box points. Logistic regression analysis was used to compare the mean dominant handgrip strength between Non-smoker, Ex-smoker, and Smoker groups.

**Figure 3:**
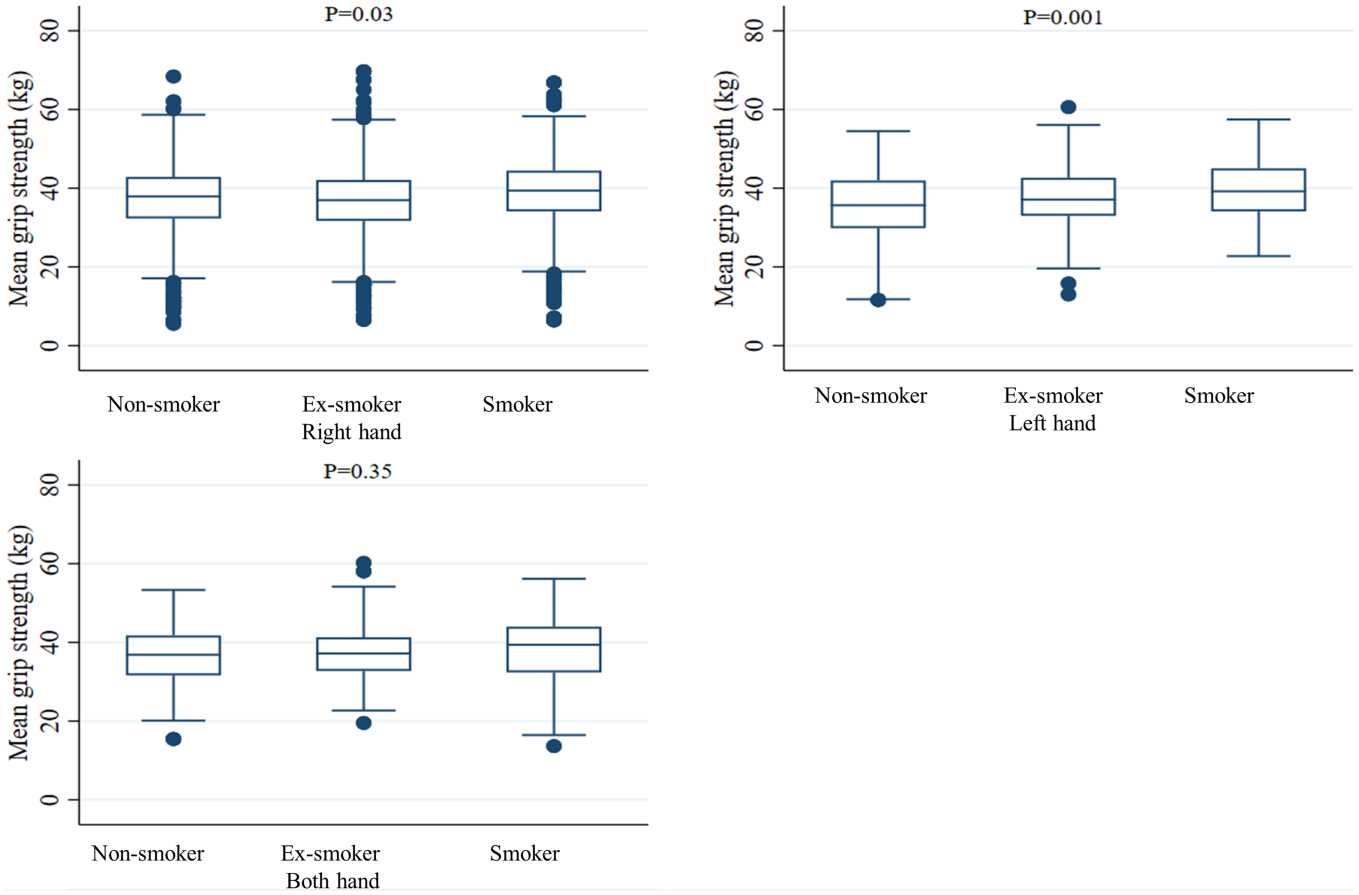
Mean dominant handgrip strength according to smoking status. The middle horizontal line represents the median level. Whiskers go from minimum to maximum values with 1st and 3rd quartile box points. Logistic regression analysis was used to compare the mean dominant handgrip strength between Non-smoker, Ex-smoker, and Smoker groups.

Smoking status was classified as current smoker, ex-smoker, or non-smoker, according to the survey results. Non-smokers included both never smokers and smokers who had smoked under 100 cigarettes in their lifetime; otherwise, they were defined as smokers. Among smokers, those who answered that they currently smoked were defined as current smokers, and those who answered that they did not currently smoke were defined as ex-smokers regardless of when they quit smoking. For the standard value of handgrip strength, we used the standard for the skeletal muscle mass (26 kg for men) suggested by the North American Foundation for the National Institutes of Health Sarcopenia Project published in 2015 (32). HGS was measured using a digital hand dynamometer (Digital grip strength dynamometer, T.K.K 5401, Japan). Trained medical technicians instructed the seated participants to hold the dynamometer with the distal interphalangeal finger joints at a 90° angle to the handle, and to squeeze the handle as firmly as they could. After participants slowly stood up, HGS was measured during expiration. A 60-s rest period was given after each HGS measurements. The average grip strength (kg) of the right and left hands was defined as mean hand grip strength (mean HGS) by measuring both hands three times each. The average of the grip force measured three times in the dominant hand (right hand, left hand, or both hands) was defined as mean dominant HGS. Grip strength was judged to be normal if it was over 26 kg and low if it was less than 26 kg. Based on previous studies, age, body mass index (BMI), household income, educational level, physical activity, alcohol consumption, presence or absence of hypertension, diabetes, dyslipidemia, and limitation of daily activities were considered as confounding variables. Correlations between each confounding variable and mean HGS and mean dominant HGS were confirmed (Figures 2, 3).

Data were obtained from the health interview survey on age, education (under high school, high school, and college or more), alcohol consumption (none, ≤1 day month, ≤2–4 days/month, ≤2–3 days/week, ≥4 days/week), and physical activity (high; those who performed high-intensity work or exercise, moderate; those who did not perform high-intensity work or exercise but did moderate-intensity work or exercise, and low; those who did not perform high-intensity work or exercise and moderate-intensity work or exercise). Ages were analyzed in groups of 19–29 years, 30–39 years, 40–49 years, 50–59 years, 60–69 years, and > 70 years. High-intensity work or exercise is a physical activity that takes a lot of breath or rapid beating of the heart for more than 10 min, such as moving heavy objects; work such as labor at a construction site; and exercise such as running, climbing, or swimming. Moderate-intensity work or exercise is a physical activity of a slight shortness of breath for more than 10 min or a slight beating of the heart, including cleaning, parenting, carrying light objects, and exercises such as fast walking, strength exercise, golf, and pilates. BMI was calculated as weight/(height)^2^ (kg/m^2^), and according to the 2018 Korean Society for Obesity Guidelines (33), <18.5 was underweight, 18.5–22.9 was normal, 23.0–24.9 was overweight, and ≥ 25.0 was classified as obese. Household income was divided into quartiles according to the standard amount by year of the survey. Diagnosis of hypertension, diabetes, dyslipidemia was defined based on the answers of self-reported questionnaire asking “Have you been diagnosed with the disease by a doctor?”(Yes/No) or “Do you take medicine or treatment for the disease?” (Yes/No). Limitation in daily activity was defined as disability in day-to-day activities such as clothing oneself or bathing alone. Hypertension was defined as total systolic blood pressure of ≥140 mmHg or diastolic blood pressure of ≥90 mmHg; dyslipidemia was defined as total cholesterol of ≥240 mg/dL, LDL-cholesterol ≥160 mg/dL, triglyceride 200 mg/dL, HDL-cholesterol <40 mg/dL; and diabetes mellitus was defined as ≥6.5% of glycated hemoglobin or fasting blood sugar ≥126 mg/dL.

### 2.3 Statistical analysis

All data were weighted by proportion for data analysis to represent the total Korean population based on the complex survey design. To investigate whether there was a difference in demographic variables according to smoking status, the chi-square test for categorical variables, which were reported as weighted percentages. The one-way of variance (ANOVA) for continuous variables were performed, which were reported as mean ± standard error (SE). A post-hoc analysis for ANOVA was performed by Bonferroni correction and the significance was based on the two-sided test value of *p* < 0.05. Multivariable logistic regression was applied and age-stratified sub-group analysis were conducted to confirm whether the mean HGS and mean dominant HGS decreased according to the smoking status. We performed unadjusted multivariate logistic regression analyses to investigate the relationship between smoking status with HGS in real setting, and adjusted analysis for age which are related with both HGS and smoking status. Finally we performed adjusted analysis for all covariates (age, BMI, household income, educational level, physical activity, alcohol consumption, hypertension, diabetes, dyslipidemia, and limitation of daily activity) which is used in previous studies. The results are presented as 95% confidence intervals (95% CI), odds ratios (OR), and adjusted OR (aOR), respectively. STATA version 16.1 (StataCorp LLC, College Station, TX, USA) was used as the analysis program. All participants gave written, informed consent, and the Institutional Review Board of the KCDC approved the study protocol for the survey. KNHANES data are publicly available and all subjects in these surveys are fully anonymized and un-identified. This study approved by the Institutional Review Board of Seoul National University Bundang Hospital (IRB No. Z-2021-162), and informed consent was waived.

## 3 Results

### 3.1 Baseline characteristics

Of the 7,649 subjects in the study, 2,669 (34.9%) were current smokers, 2,988 (39.1%) were ex-smokers, and 1,992 (26.0%) were non-smokers. Compared to non-smokers (49.2%) or current smokers (39.7%), the number of ex-smokers was smaller among those aged 19–29 years old (11.1%), higher among those aged 60–69 years old (55.7%), and higher among those aged 70 years or older (58.2%). Current smokers were the most common among those aged 30–39 (48.3%) and those aged 40–49 (41.2%) (value of *p* < 0.001). Obese people were the most common among all non-smokers, ex-smokers, and current smokers (38.2, 42.3, and 39.8%, respectively) compared to those with lower body weight, normal weight, or who were overweight (value of *p* < 0.001). In terms of household income, non-smokers, ex-smokers, and current smokers were the most common in the top 1st income quartile (32.0, 29.3 and 29.9% respectively) (value of *p* < 0.001). Regarding the physical activity, those who do not high-intensity work or exercise and moderate-intensity work or exercise accounted for the largest proportion with 1,121 (60.1%) of non-smokers, 1,789 (64.0%) of ex-smokers, and 1,597 (67.1%) of current smokers. Regarding the frequency of drinking, 624 (34.9%) non-smokers drank less than once a month, 748 (25.8%) ex-smokers drank less than 2–4 times a month, and 754 (29.0%) current smokers drank 2–3 times a week or less, which accounted for the largest proportion of each (value of *p* < 0.001). There were no significant differences about demographic and anthropometric measurements and comorbidities between non-smoker, ex-smoker, and current smokers (Table 1).

**Table 1:** Baseline characteristics of male participants according to smoking status.^1^

	Smoking status			Value of *p*
	Non-smoker (*n* = 1,992)	Ex-smoker (*n* = 2,988)	Current smoker (*n* = 2,669)	
Age (years), *N* (%)				<0.001
19–29	479 (24.1)	108 (3.6)	387 (14.5)	
30–39	370 (18.6)	298 (10.0)	624 (23.4)	
40–49	329 (16.5)	511 (17.1)	589 (22.1)	
50–59	244 (12.2)	581 (19.4)	512 (19.2)	
60–69	252 (12.6)	736 (24.6)	333 (12.5)	
≥70	318 (16.0)	754 (25.3)	224 (8.3)	
BMI^2^ (kg/m^2), *N* (%)				<0.001
<18.5	40 (2.0)	49 (1.6)	102 (3.8)	
18.5–22.9	671 (33.7)	859 (28.8)	838 (31.4)	
23.0–24.9	518 (26.1)	816 (27.3)	667 (25.0)	
≥25.0	760 (38.2)	1,262 (42.3)	1,062 (39.8)	
Household income, *N* (%)				<0.001
Low	337 (17.0)	566 (19.0)	493 (14.8)	
Mid-low	472 (23.8)	754 (25.3)	694 (26.1)	
Mid-high	539 (27.2)	787 (26.4)	777 (29.2)	
High	633 (32.0)	873 (29.3)	795 (29.9)	
Educational level, *N* (%)				<0.001
Under high school	331 (17.2)	818 (28.2)	419 (16.6)	
High school	374 (19.5)	822 (28.4)	825 (32.6)	
College or more	11,524 (63.3)	1,258 (43.4)	1,284 (50.8)	
Physical activity, *N* (%)				<0.001
High	384 (20.6)	405 (14.5)	424 (17.8)	
Moderate	360 (19.3)	601 (21.5)	358 (15.1)	
Low	1,121 (60.1)	1,789 (64.0)	1,597 (67.1)	
Alcohol consumption, *N* (%)				<0.001
None	259 (14.5)	506 (17.5)	214 (8.2)	
≤1/mo	624 (34.9)	536 (18.5)	464 (17.9)	
≤2–4/month	545 (30.5)	748 (25.8)	703 (27.0)	
≤2–3/week	262 (14.7)	693 (23.9)	754 (29.0)	
>4/week	97 (5.4)	415 (14.3)	465 (17.9)	
Hypertension, *N* (%)				<0.001
Yes	430 (21.6)	1,046 (35.0)	551 (20.6)	
No	1,562 (78.4)	1,942 (65.0)	2,118 (79.4)	
Diabetes, *N* (%)				<0.001
Yes	122 (6.1)	459 (15.4)	249 (9.3)	
No	1,870 (93.9)	2,529 (84.6)	2,420 (90.7)	
Dyslipidemia, *N* (%)				<0.001
Yes	189 (9.5)	625 (20.9)	356 (13.3)	
No	1,803 (90.5)	2,363 (79.1)	2,313 (86.7)	
Stroke, *N* (%)				<0.001
Yes	32 (1.7)	117 (4.0)	51 (2.0)	
No	1,893 (98.3)	2,790 (96.0)	2,490 (98.0)	
Limitation of daily activities, *N* (%)				<0.001
Yes	106 (5.5)	257 (8.9)	163 (6.4)	
No	1,819 (94.5)	2,647 (91.1)	2,372 (93.6)	

1 All characteristic variables were expressed as categorical variables and all are expressed as number (%).

2 BMI, body mass index.

When comparing the mean HGS of the right hand and left hand according to the smoking status, the right hand was 39.1 ± 8.0 kg in current smokers, 36.6 ± 8.3 kg in ex-smokers, and 37.0 ± 8.4 kg in non-smokers (value of *p* = 0.02). In the case of the left hand, 37.7 ± 7.4 kg in current smokers, 35.6 ± 7.9 kg in ex-smokers, and 35.7 ± 8.1 kg in non-smokers (value of *p* < 0.001), respectively. When comparing the grip strength of the dominant hand according to smoking status, when the dominant hand was recorded as the right hand, the mean HGS was 39.1 ± 8.0 kg in current smokers, 36.7 ± 8.3 kg in ex-smokers, and 37.3 ± 8.4 kg in non-smokers (value of *p* = 0.03). When the dominant hand was recorded as the left hand, the mean HGS was 39.4 ± 7.7 kg in current smokers, 36.7 ± 7.7 kg in ex-smokers, and 35.0 ± 10.0 kg in non-smokers (value of *p* < 0.001). When the dominant hand was both hands, the mean HGS was 38.5 ± 8.2 kg in current smokers, 37.4 ± 7.4 kg in ex-smokers, and 36.4 ± 7.4 kg in non-smokers, and there was no significant difference according to smoking status (value of *p* = 0.35) (Table 2).

**Table 2:** Comparison of mean hand grip strength and mean dominant hand grip strength according to smoking status.^1^

	Smoking status			Value of *p*
	Non-smoker	Ex-smoker	Current smoker	
Mean hand grip strength (kg), Mean (SD)				
Right	37.0 (8.4)	36.6 (8.3)	39.1 (8.0)	0.02
Left	35.7 (8.1)	35.6 (7.9)	37.7 (7.4)	<0.001
Mean dominant hand grip strength (kg), Mean (SD)				
Right	37.3 (8.4)	36.7 (8.3)	39.1 (8.0)	0.03
Left	35.0 (10.0)	36.7 (7.7)	39.4 (7.7)	0.001
Both	36.4 (7.4)	37.4 (7.4)	38.5 (8.2)	0.35

1 Variables are expressed as the mean ± SD (normal distribution).

### 3.2 Association between smoking status and grip strength

Table 3 shows the results of multivariate logistic regression analysis on whether grip strength decreased according to smoking status, and we divided the analysis into five models. The mean HGS of the hand with the higher average grip strength among both hands of ex-smokers and current smokers was aOR 0.61 (95% CI 0.46–0.82; value of *p* = 0.001) and 0.55 (95% CI 0.38–0.78; value of *p* = 0.001) in Model 3, respectively, showing a significantly decreased in relation to increased ratio of HGS of current smokers than ex-smokers. The aOR of mean dominant HGS between ex-smokers and current smokers was 0.78 (95% CI 0.59–1.04, value of *p* = 0.09) and 0.64 (95% CI 0.46–0.91, value of *p* = 0.01), compared to non-smokers, respectively. Although the mean dominant HGS was not significantly lower in ex-smokers, the mean dominant HGS was significantly higher in current smokers than that in non-smokers.

**Table 3:** Odds ratio of hand grip strength according to smoking status.

	Smoking status		
	Non-smoker	Ex-smoker	Current smoker
Mean HGS			
Model 1 (95% CI)	1 (Ref)	0.90 (0.73–1.11)	0.43 (0.33–0.56)
Model 2 (95% CI)	1 (Ref)	0.55 (0.43–0.70)	0.61 (0.46–0.80)
Model 3 (95% CI)	1 (Ref)	0.61 (0.46–0.82)	0.55 (0.38–0.78)
Mean dominant HGS			
Model 1 (95% CI)	1 (Ref)	1.02 (0.83–1.24)	0.48 (0.38–0.62)
Model 2 (95% CI)	1 (Ref)	0.63 (0.50–0.79)	0.68 (0.52–0.89)
Model 3 (95% CI)	1 (Ref)	0.78 (0.59–1.04)	0.64 (0.46–0.91)

Model 1 unadjusted; Model 2 adjusted for age; Model 3 adjusted for age, BMI, household income, education level, physical activity, alcohol consumption, hypertension, diabetes mellitus, dyslipidemia, stroke, limitation of physical activities.

HGS, handgrip strength; CI, confidence interval; Ref, reference group.

Multivariate logistic regression analysis was performed to determine whether the grip strength decreased according to smoking status, stratified by age. Adult male current smokers aged 60–69 and 70 years or older had significantly decrease in association of mean HGS increase than non-smokers (aOR = 0.31, 95% CI 0.11–0.90, aOR = 0.43, 95% CI 0.26–0.72, respectively). The mean dominant HGS increase was also significantly higher decreased odds than that of non-smokers in the age group of ≥70 years (aOR = 0.50, 95% CI 0.30–0.85; value of *p* = 0.01). In other age groups, there was no significant difference in the mean HGS and the mean dominant HGS according to smoking status (Table 4).

**Table 4:** Odds ratio of handgrip strength according to age.^1^

	Mean (SD)	Unadjusted			Adjusted		
		Non-smoker	Ex-smoker	Current smoker	Non-smoker	Ex-smoker	Current smoker
<40							
Mean HGS	41.5 (7.3)	1 (Ref)	0.08 (0.01–0.60)	0.36 (0.18–0.74)	1 (Ref)	0.14 (0.02–1.20)	0.58 (0.22–1.51)
Mean dominant HGS	40.5 (8.1)	1 (Ref)	0.06 (0.01–0.45)	0.38 (0.20–0.70)	1 (Ref)	0.13 (0.02–1.01)	0.66 (0.31–1.42)
40–49							
Mean HGS	41.9 (6.9)	1 (Ref)	0.32 (0.11–0.93)	0.33 (0.12–0.91)	1 (Ref)	0.42 (0.12–1.53)	0.37 (0.09–1.53)
Mean dominant HGS	40.8 (8.2)	1 (Ref)	0.64 (0.22–1.86)	0.56 (0.20–1.62)	1 (Ref)	1.60 (0.38–6.69)	0.88 (0.17–4.45)
50–59							
Mean HGS	39.7 (6.2)	1 (Ref)	0.41 (0.14–1.19)	0.47 (0.16–1.35)	1 (Ref)	0.41 (0.11–1.46)	0.50 (0.14–1.77)
Mean dominant HGS	38.4 (7.6)	1 (Ref)	0.27 (0.10–0.78)	0.77 (0.33–1.80)	1 (Ref)	0.23 (0.07–0.79)	0.55 (0.19–1.62)
60–69							
Mean HGS	36.5 (6.3)	1 (Ref)	0.65 (0.38–1.21)	0.41 (0.18–0.94)	1 (Ref)	0.72 (0.33–1.56)	0.31 (0.11–0.90)
Mean dominant HGS	35.3 (7.4)	1 (Ref)	0.66 (0.38–1.12)	0.54 (0.28–1.05)	1 (Ref)	0.82 (0.42–1.59)	0.51 (0.22–1.18)
≥70							
Mean HGS	29.9 (7.1)	1 (Ref)	0.63 (0.47–0.84)	0.84 (0.58–1.22)	1 (Ref)	0.57 (0.39–0.83)	0.43 (0.26–0.72)
Mean dominant HGS	28.5 (8.1)	1(Ref)	0.77 (0.57–1.03)	0.86 (0.59–1.27)	1(Ref)	0.77 (0.53–1.12)	0.50 (0.30–0.85)

^1^Adjusted for age, BMI, household income, education level, physical activity, alcohol consumption, hypertension, diabetes mellitus, dyslipidemia, stroke, limitation of physical activities. HGS, handgrip strength; SD, standard deviation; CI, confidence interval; Ref, reference group.

## 4 Discussion

This study investigated the relationship between smoking and grip strength in 7,649 adult men aged ≥19 years who participated in the KNHANES. In this study, the mean hand grip strength of current smokers and ex-smokers was found to be stronger than that of non-smokers, contrary to the expectation that grip strength would be significantly weaker in current smokers and ex-smokers than in non-smokers. This result was the same when analyzed after adjusting for factors expected to affect grip strength, including age, BMI, household income, education level, physical activity, alcohol consumption, hypertension, diabetes, dyslipidemia, and limitations of physical activities. In addition, the mean HGS of current smokers was stronger than that of ex-smokers and non-smokers in the 60–69 and ≥ 70 years age groups.

There have not been many studies performed on the relation of sarcopenia measured by HGS and diverse health factors yet. In the previous study, when analyzing some groups of smokers, it was confirmed that the grip strength of current smokers decreased compared to that of non-smokers, which is contrary to this study on the relationship between grip strength and smoking. Smoking was associated with sarcopenia in non-obese subjects but not significantly associated with sarcopenia in obese subjects in a 2019 cross-sectional study that analyzed the relationship between smoking status and sarcopenia according to obesity in 9,385 middle-aged people from 2008 to 2011 (20). However, our study was conducted only on male adults over the age of 19 and adjusted BMI, so it is different from the existing study. An Indian study of athletes between the ages of 19 and 30 years showed a decrease in muscle strength in current smokers compared with non-smokers (34). Since the study analyzed athletes who are very young and have better muscle strength than ordinary adults, various diseases or health problems affect muscle strength can be very different from the general population. There are several studies that support our findings, which were retrospective studies using previous data. In a study on grip strength according to the smoking status of Korean adults using the 7th KNHANES for 9,848 people, the mean HGS and the mean dominant HGS were stronger in current smokers and ex-smokers than that in non-smokers (value of *p* < 0.001) (7). However our study has the advantage of analyzing participants for 3 years, adjusted for all comorbid diseases such as hypertension, diabetes mellitus, dyslipidemia, and stroke, and included a wider range of subjects than above study. Additionally, in a study on the relationship between smoking status and grip strength in adults over 50 years of age using data from the US Health and Retirement Study from 2012 to 2014, there was no significant relationship between grip strength in both current smokers and ex-smokers when it was adjusted for gender, age, race, comorbidity, educational level, house income, depression, cognitive function, and physical activity (value of *p* = 0.62, 0.17, respectively) (18). However, the subject of study was older than our study respectively, so they could vulnerable to smoking than younger subjects.

Sarcopenia is associated with the internal environment of the organism and results from endogenous influences such as hormone, pro-inflammatory cytokines, a reduction in the number of motor units, and insulin resistance with aging (19). Additionally, there are external risk factors including poor nutrition, decreased physical activity, alcohol consumption and cigarette smoking (19). For instance, a study on the relationship between smoking and the incidence of sarcopenia found that smoking significantly increased the risk of developing sarcopenia over a 5-year follow-up aged over 65 years (35), but smoking status was not associated with sarcopenia in over 50 years of age men and women, and smoking level was not associated with sarcopenia in men but associated with sarcopenia in women (36). Therefore sarcopenia is influenced by various factors, and our study could not account for all of these multifactorial influences due to the limitations of the retrospective design, which involved reviewing previous cross-sectional surveys. Another possibility is that nicotine in tobacco smoke may have immediate beneficial effects on motor skills, and the lingering effects of nicotine or similar mediators could impact the relationship of smoking status and HGS (37). Furthermore, it is possible that individuals who were already in poor health may quit smoking, or unhealthy subjects may have been less likely to respond to the survey (38).

This study had several limitations. First, as this was a retrospective data review study using previous cross-sectional survey, it is possible that individuals categorized as non-smokers or ex-smokers did not smoke or stopped smoking due to existing health problems. Notably after adjusting for confounding variables according to age, the mean HGS of current smokers was found to be stronger only in individuals over 60 years of age, as shown in Table 4. This makes it possible to hypothesize that people who smoke at an older age have relatively fewer health problems. Second, a person marked as non-smoker may be an ex-smoker actually. Due to the limitations of the questionnaire, it may be difficult to distinguish between ex-smokers and non-smokers (39). Third, most of the non-smokers were relatively young, and there were few people with grip problems in the younger age group; therefore, it was not possible to separate them during the analysis. As shown in Table 1, non-smokers were young and ex-smokers were older; therefore, in the overall analysis, the effect of age on grip strength may remain despite correction for the effect of age. To correct for this, grip strength by age was analyzed, but the correction may not be sufficient; further analysis with a matched sample in a future study will be necessary. Fourth, since the people who participated in the KNHANES are relatively healthy people living in the local community, it is possible that people with advanced sarcopenia were excluded from the sample because they were in nursing homes. Fifth, research and adjustments were not made on study participants with pain in the fingers, hands, or wrists. Participants who answered that they had osteoarthritis or rheumatoid arthritis were analyzed without considering the presence or absence of osteoarthritis or rheumatoid arthritis as it was difficult to know where the affected part was because the questionnaire was not completed. Nevertheless, this study is meaningful in that it confirmed the association between smoking status and grip strength on a large scale in Korean adult males. Smoking adversely affects health, and grip strength is an indicator of overall body strength. There are limitations in analyzing the effects of smoking on HGS through cross-sectional studies, because some smokers quit due to health issues. However, it should be noted that clinically, non-smokers have a lower HGS and are more likely to be frail than current smokers. It is possible that clinically unhealthy people have already quit smoking, so the smoker’s grip may have been strong. In particular, in old age, people who have already quit smoking can be frail, therefore a non-smoker should not conclude that there is no frailty.

## 5 Conclusion

In our study, the mean HGS of current smokers and ex-smokers was found to be stronger than that of non-smokers after adjusting for factors related to grip strength. By age group, current smokers aged 60–69 years and older than 70 years had stronger mean HGS than ex-smokers and non-smokers in the same age group. Although it is generally thought that nonsmokers are healthier, it should be highlighted that nonsmokers may actually have lower muscle strength. Therefore, it is necessary to rethink the perception of nonsmoker’s health, to tailor interventions for older smokers, and understand the impact of smoking on muscle strength. A causal relationship between smoking status and HGS could not be definitively identified and still inconclusive owing to the retrospective data review study design using previous survey data. However this study has significance since it used a large-scale survey to confirm the association between smoking and grip strength Therefore, longitudinal studies will still be required to how the cigarette smoking contribute to grip strength and to understand the association between smoking status and grip strength.

## Data availability statement

The datasets presented in this study can be found in online repositories. The names of the repository/repositories and accession number(s) can be found below: https://knhanes.kdca.go.kr/knhanes/sub03/sub03_01.do.

## Author contributions

HL and HS: conceptualization. J-RL: methodology. HS and J-RL: data collection and investigation. EC and HS: writing—original draft preparation. EC: writing. All authors contributed to the article and approved the submitted version.
